# Energy-Efficient LoRa Routing for Smart Grids

**DOI:** 10.3390/s23063072

**Published:** 2023-03-13

**Authors:** Raja Kishore Repuri, John Pradeep Darsy

**Affiliations:** School of Electronics Engineering, VIT-AP University, Amaravati 522237, Andhra Pradesh, India; rajakishore.20phd7039@vitap.ac.in

**Keywords:** advanced metring infrastructure, cluster head selection, energy efficiency, IoT, LEACH, long range (LoRa), network lifetime, smart grid, WSN, optimisation

## Abstract

Energy-efficient routing protocols in Internet of Things (IoT) applications are always of colossal importance as they improve the network’s longevity. The smart grid (SG) application of the IoT uses advanced metring infrastructure (AMI) to read and record power consumption periodically or on demand. The AMI sensor nodes in a smart grid network sense, process, and transmit information, which require energy, which is a limited resource and is an important parameter required to maintain the network for a longer duration. The present work discusses a novel energy-efficient routing criterion in an SG environment realised using LoRa nodes. Firstly, a modified LEACH protocol–cumulative low-energy adaptive clustering hierarchy (Cum_LEACH) is proposed for cluster head selection among the nodes. It uses the cumulative energy distribution of the nodes to select the cluster head. Furthermore, for test packet transmission, multiple optimal paths are created using the quadratic kernelised African-buffalo-optimisation-based LOADng (qAB_LOADng) algorithm. The best optimal path is selected from these multiple paths using a modified version of the MAX algorithm called the SMAx algorithm. This routing criterion showed an improved energy consumption profile of the nodes and the number of active nodes after running for 5000 iterations compared to standard routing protocols such as LEACH, SEP, and DEEC.

## 1. Introduction

Smart grids (SGs) have efficiently tackled the issue of electricity wastage by addressing problems such as inefficient consumer electronics, a lack of accountable technology, sporadic monitoring and communication, and the absence of an electricity storage system [1]. The utilities in SGs are permitted to employ information and communication technologies (ICTs) to assess, monitor, and regulate power supply and demand with little negative environmental impact [2]. By offering two-way communication protocols, IoT-inspired applications provide significant solutions to improve power supply and demand concerns by assisting in the modernisation of outdated power grids into SGs [3]. Globally, the number of IoT devices installed has increased, and the electricity grid is no exception. These IoT devices are a collection of specialised physical items with embedded technology that can communicate with the outside world. In order to monitor and manage infrastructure, many IoT devices are integrated into SGs. These devices autonomously log the grid condition information and communicate to the entities, which use these data to manage the grid properly [4].

Communication technologies play a crucial role in the development and operation of SGs [5,6]. They enable the exchange of data and information between different components of the grid. Various architectures and frameworks have been developed to aid in the selection of communication technologies for SGs [7,8], such as the analytic hierarchy process (AHP) and multi-criteria decision analysis (MCDA). These models consider various factors and help decision-makers select the most appropriate communication technology for their specific smart grid project. Some common communication technologies and standards [9] used in smart grid systems include advanced metring infrastructure (AMI), supervisory control and data acquisition (SCADA), distributed energy resource management systems (DERMSs), OpenADR, IEEE 802.15.4, IEC 61850, and ZigBee. These technologies and standards are designed to be energy-efficient, reliable, and secure and are constantly evolving to improve the operation of smart grids. Efforts towards selecting the shortest path [10,11] or optimal path for communication technology in smart grids involve analysing various factors such as reliability, security, scalability, and cost-effectiveness. The selection of communication technologies for smart grids must also consider the specific requirements of the grid, such as the type and number of devices to be connected, the distance between devices, and the bandwidth required for data transmission. The Internet of Things based communication architecture enables the development and operation of interoperable microgrids, which include the energy management and switch port security aspects, to reduce the burden on the utility grid [12].

**Figure 1:**
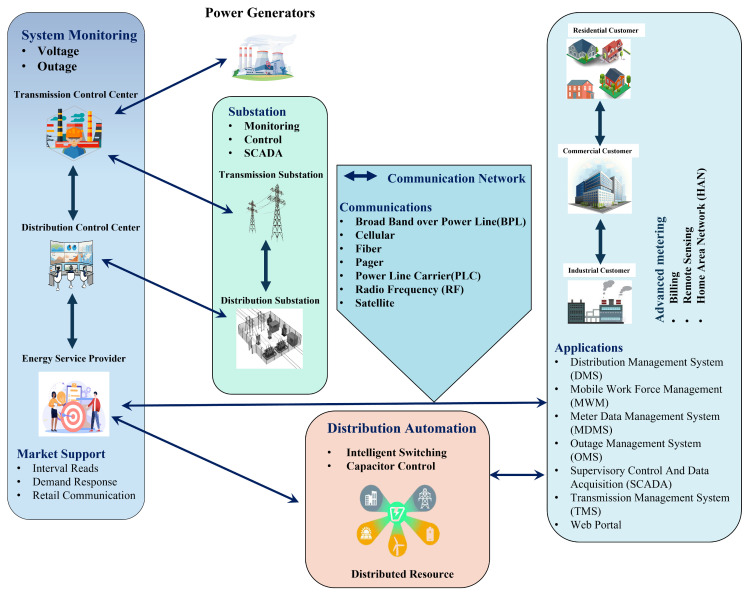
Concept of the smart grid with its components.

Figure 1 shows the different components of the smart grid (SG) and how they communicate. The significant components are advanced metring infrastructure (AMI), advanced communication networks, distribution automation (DA), energy storage systems (ESSs), transmission, distribution subsystems, and control centres. To make smart grid technology work, there must be advanced communication networks for data transmission between the utility and the consumer, including fibre optic cables, wireless networks, or satellite communications. This work focused on the communication between the AMI and LoRa gateways.

The wireless sensor networks (WSNs) utilised in an SG allow sensors to gather data autonomously and efficiently route packets to the BS. Each node in an SG forms a WSN-IoT network, which then gathers information from a specified area and forwards it to the destination. Such a data detection and routing technique requires extensive information exchange between nodes, resulting in more energy reduction [13].

Forming a separate sensor network requires the random placement of a certain number of multi-functional sensor nodes throughout a remote region, which have finite battery power [14]. Low-power WSNs are the focus of most modern sensor-aided systems, which have also transformed industrial monitoring and diagnostics. These weakly powered nodes can communicate wirelessly and form a network quickly. Because WSNs are often put in unmanaged, difficult environments, retrieving batteries when they run out of power is challenging. Power quality and network lifetime are prioritised to improve energy-constrained WSNs. The best approach for sustaining the energy consumption in sensor nodes is thought to be clustering techniques [15]. Each cluster has a single cluster head (CH) and a few cluster member (CM) nodes. The CM sends the information to its corresponding CH in the data aggregation cluster and transfers it to the base station (BS). If a node runs out of energy during a round, then it is declared as dead, and the network is said to have come to an end. Delivering the combined data from all CHs to the BS for additional analysis is the goal of a WSN protocol. This kind of routing results in high energy costs and unexpected node deaths, both of which can potentially render the network inoperable; hence, it is essential to find ways to extend the network’s lifetime effectively. Under adverse wireless communications situations, providing quality-aware data delivery such as scalability, network coverage, throughput, stability, lifetime, reliability, complexity, and latency comprises the critical design concerns in SGs [16].

Long range (LoRa) is one of the most-promising large-scale smart grid communication platforms. LoRa is a low-energy wide-area network specifically suited to transmitting sensor data [17]. LoRa technology allows power station sensors and controllers to communicate data to a monitoring centre through walls, buildings, and other obstacles. Thus, it requires fewer gateways than other wireless technologies. Because of the possibly complicated environment in which the sensor devices work, issues occur when deploying LoRa technology in dense and significantly varying wireless settings [18]. To solve these difficulties, hierarchical routing and sensor node clustering have been proven to extend the network lifetime [19]. Hence, this paper employed an energy-efficient LoRa smart grid system using optimal CH selection and path selection techniques. We can also apply the proposed method by considering the node as the energy router, which is primarily a routing device within a microgrid. At the same time, an energy hub is a more comprehensive energy management platform, which integrates multiple energy systems and microgrids [20].

### 1.1. Contribution

The work presented in this paper makes the following contributions:

- A modified LEACH (Cum_LEACH) protocol based on the cumulative energy distribution is proposed for CH selection.
- Multiple paths are created using a novel method, quadratic-kernel-exploitation-based African buffalo optimisation (QKE-ABO), which transmits the data from the transmitting node to the receiving node when they are far away from each other.
- A modified Mexican axolotls optimisation, scrambled mutation Mexican axolotls optimisation (SMax), is proposed for multi-path scenarios.

**Figure 2:**
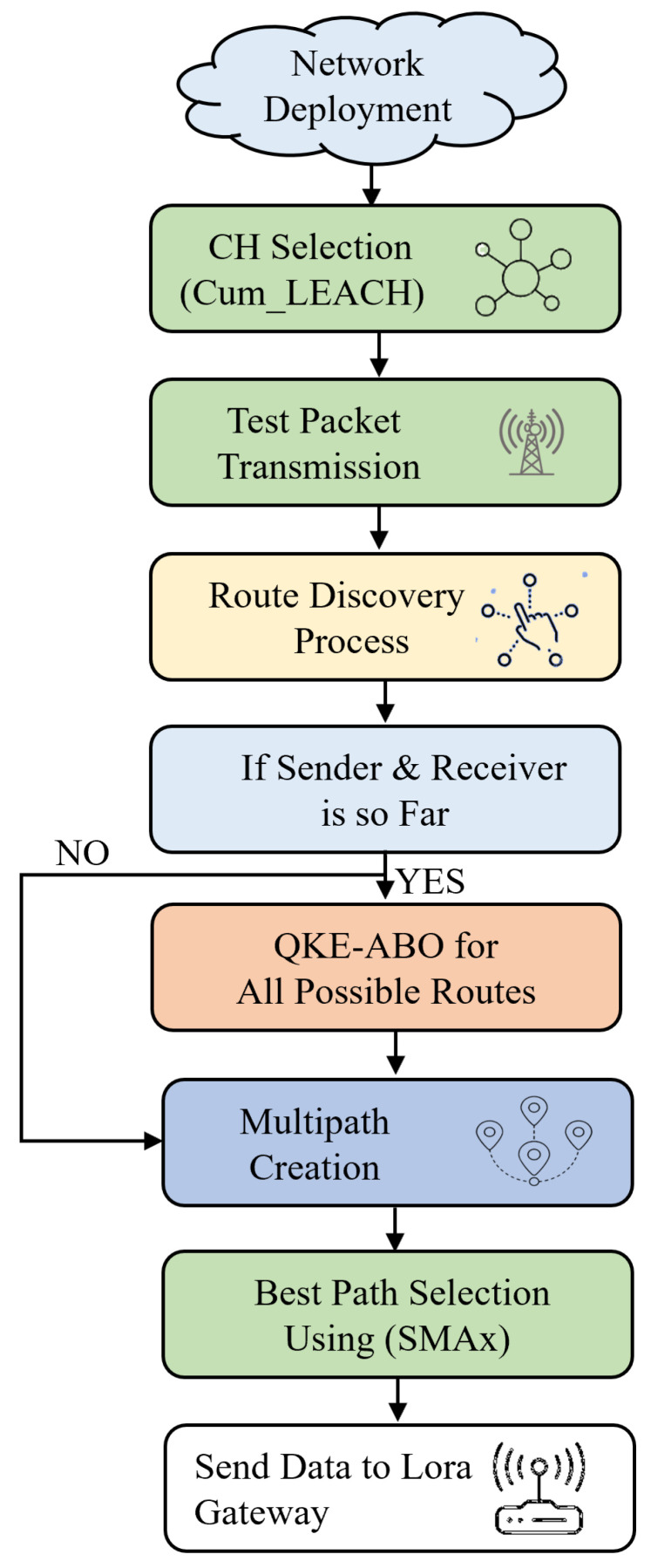
Flow diagram of the different phases of the proposed system.

This paper analysed the effectiveness of our proposed optimal path algorithms from different aspects by conducting extensive simulation tests using the MATLAB simulator. The different phases involved in the proposed system are shown in Figure 2: initially deploying the sensor node, then using Cum_LEACH to select the CH, followed by test packet transmission if the transmitting and receiving nodes are very far from each other using a QKE-ABO optimisation technique to create multiple paths, then from these paths, using SMAx to choose an optimised path. The detailed flow is explained in Section 3. The phases of the communication presented in this new contribution are given in Figure 2.

### 1.2. Paper Structure

This paper is organised as follows: Section 2 discusses various CH selection methods and optimal path selection techniques in the literature. In Section 3, the proposed CH selection method, Cum_LEACH, and optimisation techniques, the QKE-ABO and SMAx algorithms, are discussed. The simulation outcomes of the proposed work and their discussion are presented in Section 4. Lastly, in Section 5, the conclusion of the work is discussed.

## 2. Literature Review

Much research has been conducted recently on taking advantage of the fast transfer rate and low latency offered by modern communication technologies. Smart grids (SGs), on the other hand, are widely installed by utility companies because their advanced metring infrastructure (AMI) provides various benefits, including cost savings for metre reading and service team deployment. AMIs can also be installed and give coverage anywhere there is an energy consumer, even in rural areas. It is also critical to use an adequate wireless communication device to meet the demands of SG operation. Communication technologies such as Bluetooth, ZigBee, and WiFi are well-developed for IoT applications, but they are limited to a specific range and waste much power. Thus, they are not suitable for transmitting data in the SG or in other wide-area applications [21]. The protocol for precision agriculture (PA) systems utilises both WiFi and LoRa wireless technologies, where the CH node is the bridge that allows communication between them. A tree topology of multiple hops has been introduced, which enables reaching further distances while reducing data transmission from one node to another [22].

To meet the needs of the SG to save power and send data over long distances, a new infrastructure for wireless communication called the low-power wide-area network (LPWAN) is being built. LPWANs [23] assist with the transmission distance–energy efficiency trade-offs. Currently, LoRa is one of the most-popular LPWAN technologies, and the performance of the LoRa modules can be improved by customising the parameters, including the bandwidth, transmission power, code rate, and spreading factor (SF) [24,25]. Integrated home automation systems that utilise multiple communication technologies such as LoRaWAN, server-based LoRa gateways, and Bluetooth connectivity to control different types of appliances were proposed by [26]. IoT-based AMI daily reporting and billing in a residential grid using LoRa technology were designed by [27]. A new two-hop implementation of the LoRa WAN LEACH protocol (LLP) was proposed by [28] using the LEACH algorithm to optimise the throughput and power dissipation. Table 1 compares some of the energy-efficient routing protocols used for SG communication in WSNs.

In homogeneous sensor networks, LEACH [36] is a self-organising, flexible clustering approach using randomisation to distribute the energy load uniformly throughout the network. In LEACH, grouped nodes can be clustered, with one node serving as the local base station (BS) or cluster head (CH). Sensors choose to be local cluster heads with a certain probability at any given time. The cluster head communicates its status to other nodes in the network. LEACH alternates the function of the CH among all nodes to evenly share the energy load. Even if the network is capable of adjusting and reducing the protocol’s performance, a poor clustering design will still affect its operation if the CHs are not optimally selected for the frequent rounds. Therefore, the most-challenging aspect of dynamic clustering is CH selection [37]. Every time a CH is re-selected, the priority for CH selection causes dynamic clusters to be formed, which increases the energy overheads.

An upgraded version of LEACH, designated LEACH-C [38], allows the formation of clusters using a centralised clustering method. LEACH-C improves the network performance by distributing CHs around the network to build better clusters. The probability calculation takes into account the residual energy of the nodes. Thus, the nodes with higher energy have a greater chance of being picked as CHs. The modified LEACH protocol [39] is a well-known WSN clustering protocol. The basic idea is to make the clustering process dynamic by randomly selecting the CH and allowing the nodes to join the CH that is geographically nearest to them. The chosen CHs form the basis of this network’s topology, which is inefficient since it ignores the residual energy at each node. A novel two-CH method for minimal energy consumption was proposed by [40] in that one CH lies closer to the BS and the other CH is selected randomly depending on the energy constraints. The randomly selected CH transmits the data to the nearby BS. As a result, the two CHs ensure the minimum energy loss in the network.

Heterogeneous-aware routing protocols such as the stable election protocol (SEP) and distributed energy-efficient clustering (DEEC) have been introduced. The SEP [41] comprises heterogeneous nodes to boost the network’s stability and longevity. The SEP has two types of nodes: normal nodes (NNs) and advanced nodes (ANs). Among these nodes, the AN acts as a CH because it has a higher estimated energy than the NN, stabilising the energy between CH and non-CH nodes. The hotspot problem is one problem with direct communication. This happens because the CH to the sink node uses much energy, which affects the lifetime of the network. The SEP functions similarly to LEACH and does not account for residual energy in determining the CH rotating process or election probability, which are directly tied to the node’s initial energy. DEEC [42] is used for heterogeneous WSN applications. As opposed to the SEP, in DEEC, the ratio between each node’s remaining energy and the network’s average energy is considered for the CH selection. The remaining energy of the nodes in the network may not be uniform. Sensor nodes with the maximum residual energy have a better probability of becoming CHs when compared to other nodes. This gives a more excellent performance for the residual energy, node lifetime, and network lifetime than the SEP and LEACH in a heterogeneous WSN with a multi-level environment. DEEC cannot be used when the BS is placed far from the sensor nodes since it assumes the BS is positioned in the middle of the WSN. The enhanced DEEC proposed by [43] balances the WSN energy and extends its lifetime using a priority queue.

In a smart grid communication network, a WSN has been considered in several works. For smart homes and cities, smart metring, and smart markets, the wireless technologies used can be integrated with a WSN for multipath routing [44]. Reference [45] presented an energy-efficient multi-hop LEACH (EEM-LEACH) protocol, which allows sensor nodes to send the data directly to the BS if the distance between the sensor nodes is less than that of the CHs. The EEM-LEACH protocol can be applied efficiently in small areas. However, if the source and destination nodes are far apart, the data are transmitted with the help of intermediate devices, and for this purpose, the lightweight on-demand ad hoc distance vector routing (LOADng) protocol was proposed, which works efficiently in a low-power noisy environment. The LOADng algorithm has a reactive functionality, which builds a path between the nodes only when the data packets need to be exchanged. As a result, a source (SRC) node that wishes to send a data packet must initiate a route discovery process to locate and build a path to the destination (DEST) node. However, this algorithm is affected by route construction delays and difficulties caused by inefficient flooding, which consumes much energy, with much overhead and many packet collisions [46]. Based on LOADng’s restrictions in MP2P settings [47], a collection tree extension for the protocol has been proposed. This proposal, known as LOADng-CTP, adds proactive capabilities to LOADng and assists nodes in creating a routing tree to send data packets from the source node to the destination node. The performance of LOADng-CTP and RPL was also evaluated in an AMI [48]. In addition, the performance of LOADng with various routing metrics was investigated in [49].

In this work, the Cum_LEACH protocol was used for CH selection, whereas the LOADng algorithm was used to transmit the data between the CH and the BS when the distance between them is large. There may be multiple paths existing between the CH and BS when the distance between them is very large. Therefore, this requires using an optimisation algorithm in conjunction with the LOADng algorithm, which is a path planning algorithm. Below are a few cases of optimisation techniques used along with LEACH to find the optimal path.

Particle swarm optimisation (PSO) [50] optimises the LEACH protocol, most commonly used in WSN routing protocols. Here, it was used for optimal communication paths in the LEACH protocol. PSO defines the optimal CH location by optimising an objective function based on the node degree, headcount, residual energy, and intra-cluster distance of the possible CH [51]. Centralised-PSO [52] is a new clustering algorithm based on PSO developed for CH selection by formulating the CH selection problem as a single-objective optimisation problem. This objective function considers the residual energy of each sensor node when each CH is close to other sensor nodes.

In the present work, African buffalo optimisation (ABO) [53] was used in association with the LOADng algorithm, called QKE-ABO, to find the optimal path to transmit the data between the CH and BS, and it was compared with other existing optimisation techniques such as the pelican optimisation algorithm (PAO) [54] and the ant lion optimisation method (ALO) [55] in terms of the convergence value for the optimal solution. To discover multiple routing paths in between the source node and the destination node, the routing path must be consistent, have high energy efficiency, and be the shortest to obtain the minimum transmission delay and energy consumption. In an SG communication network, an essential area to be covered is choosing the appropriate routing path. By utilising the SMAx algorithm, the optimal routing path is discovered and compared with the sparrow search algorithm (SSA) [56] and Mexican axolotls optimisation (MAX) [57].

## 3. Proposed IoT-Based Communication in Smart Grid Environment

**Figure 3:**
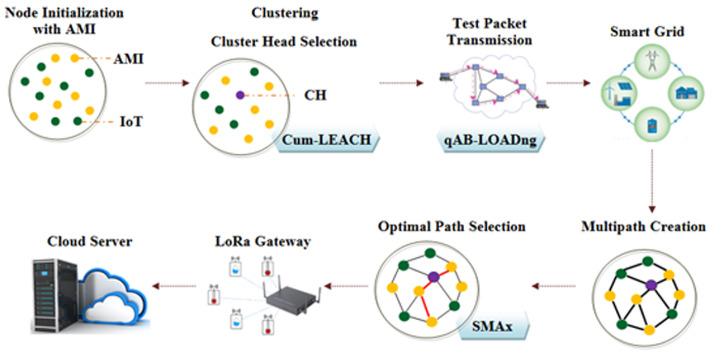
Workflow of WSN communication with proposed techniques at various stages.

This paper proposes an energy-efficient routing and optimisation technique for smart grid communication through LoRa. The model comprises six phases: node initialisation, clustering, test packet transmission, multi-path creation, and optimal path selection; then, the data are transmitted through LoRa gateways. The workflow used in the present work is given in Figure 3.

### 3.1. Node Initialisation

Initially, the sensor nodes are initialised for data transmission. The main task of a sensor node is to collect data from the sensing domain and send them to the BS through multi-node communication. The nodes are clustered to ensure the availability of the minimum energy required to transmit the data and maintain the energy balance in the network. The nodes are assigned randomly in a pre-defined area. The notations used in the Cum_LEACH CH selection and the optimisation techniques are shown in Table 2. The N nodes are initialised in Equation (1), where the set of sensor nodes is specified by *ℵ*, and the ith sensor node is denoted as ${ℵ}_{i}$.

(1) $$ℵ=\left\{{ℵ}_{i}\right\},i=1,2,3,\ldots \ldots ,N$$

### 3.2. Clustering

In this phase, the sensor nodes *ℵ* are grouped into clusters using the cumulative-distribution-based low-energy adaptive clustering hierarchy (Cum_LEACH) algorithm. This clustering algorithm helps to improve the lifetime and efficiency of the network, which performs the energy load distribution equally among all the nodes of a cluster with two significant stages, namely setup and steady-state. In the first phase, the formation of the clusters and the electing of the CHs are performed, and then, the data transfer to the BS occurs in the second phase. The LEACH algorithm uses a randomised polling approach to select the CHs to ascertain the energy consumptionamong the network nodes. As the CH selection is random, the LEACH protocol does not ensure uniformity in CH placement throughout the network. There is, thus, the possibility that the CHs will be concentrated in certain areas and may not be present in some other areas. Therefore, a cumulative distribution approach for selecting the CH is proposed.

1. Cum_LEACH cluster head selection: CHs are responsible for collecting the data from all the nodes of their own clusters and transmitting the information to the BS. This reduces the number of transmissions to the BS and ensures a balanced energy utilisation of all nodes and a more extended network lifetime. Each node participating in the CH selection process is assigned a random value *ℓ* between 0 and 1. The probability of a node being selected as a CH is defined using a uniform probability distribution function. This is calculated using the cumulative distribution function given in Equation (2).
  (2) $$\Omega_{cdf(\ell )}=\alpha \left(x\leq \ell \right)$$
  where $\Omega_{cdf}$ is the cumulative distribution function, α is the probability, and *x* is the random variable indicating the nodes. In the CH selection phase, each node must calculate the probability threshold value using Equation (3). The node elects itself to become the CH when the cumulative probability function is smaller than the threshold.
  (3) $$\lambda \left(i\right)=\frac{c}{1-c\left(r_{cu}\right)\;\mathrm{mod}\;\left(\frac{1}{c}\right)},\;i\in^{g}{ℵ}_{i}$$
  where ${\;}^{g}{ℵ}_{i}$ is the set of nodes that do not participate as CHs in the former rounds and $r_{cu}$ is the current round. If the ith node is not selected as a CH in the last (1/*c*) rounds, then $i\in {\;}^{g}{ℵ}_{i}$, as shown in Equation (3); the threshold correlates not only with *c*, but also with the remainder from dividing round *r* by [1/*c*]. As a result, if node (*i*) was not a CH in the previous [1/*c*] round, the threshold value will gradually increase. The condition for a node to be nominated as the cluster head is then specified in Equation (4).
  (4) $${ℵ}_{CH(i)}=\left\{\begin{array}{cc} {ℵ}_{i}=1, & \;\mathrm{if}\;\left.\Omega_{cdf(\ell )}<\lambda \left(i\right)\right.\\ {ℵ}_{i}=0, & \;\mathrm{otherwise}\; \end{array}\right.$$
  where ${ℵ}_{CH(i)}$ denotes the number of CHs selected. Once the node is selected as a CH, it cannot become a CH again until all the nodes of the cluster have become cluster heads once. In this way, each node is given an equal opportunity to become a cluster head and an equal energy dissipation throughout the sensor nodes.
2. Set-up stage: After the selection phase, the announcement of CHs is made by the CH node broadcasting a small-sized message called an advertisement message (ADV) to all other non-CH nodes to form clusters. Each node selects the cluster head that is closest to them for communication regarding the received signal strength. A message is transmitted to that CH to be a member of a cluster when the node chooses its potential CH. The acknowledgements and time-division multiple access (TDMA) schedule are relayed by the CH node to its cluster member nodes. To avoid collisions, a time slot is allotted by TDMA to each sensor node.
3. Steady-state: Using TDMA, the sensed data are sent by the SN to the CHs in their time slots assigned by the CHs in steady-state usage. The CH collects the data and transmits them to the BS. In the process of transmitting the test packets to the destination, if the source and destination are separated by a huge distance, the data are transmitted via intermediate devices between the source and the destination. For that purpose, the optimal lightweight on-demand ad hoc distance vector routing (LOADng) protocol was used. In the case of a nearby destination, the proposed system creates multiple routing paths between the nodes.

### 3.3. Test Packet Transmission

**Figure 4:**
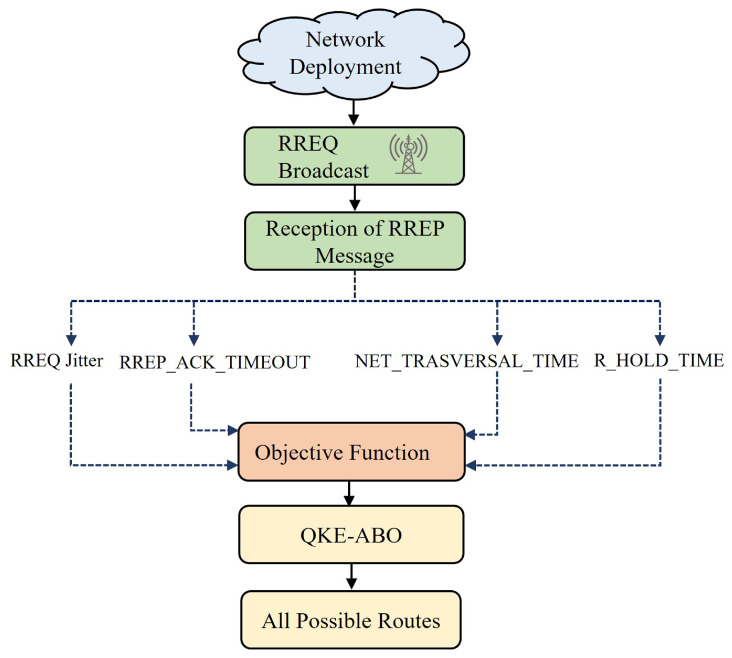
Route discovery process of the QKE-ABO algorithm.

The path among the nodes is created on demand in a reactive route discovery mechanism of LOADng, a routing protocol. Only when the data packets need to be transferred is a route among the nodes constructed by the LOADng protocol in the routing process. To find a route to the intended destination, a source (SRC) node that wishes to transmit a data packet must start the route discovery process (RDP) by broadcasting a route request (RREQ) message. Message processing is executed by every node that receives an RREQ, along with considering the message to be forwarded. Until the RREQ arrives at the desired location, this procedure continues. The network efficiency is reduced because the elements affecting the network efficiency are not taken into account while selecting a transmission path. To identify all the possible routes for data transmission, a metaheuristic algorithm that optimises the parameters such as R_HOLD_TIME, RREQ jitter, NET_TRANSVERSAL_TIME, and RREP_ACK_TIMEOUT was incorporated into the proposed protocol. By utilising the quadratic-kernel-exploitation-based African buffalo optimisation (QKE-ABO) algorithm, the parameters were chosen optimally to improve the LOADng protocol’s performance. The route discovery process of the QKE-ABO algorithm is shown in Figure 4.

#### Parameter Optimisation Using QKE-ABO Algorithm

**Figure 5:**
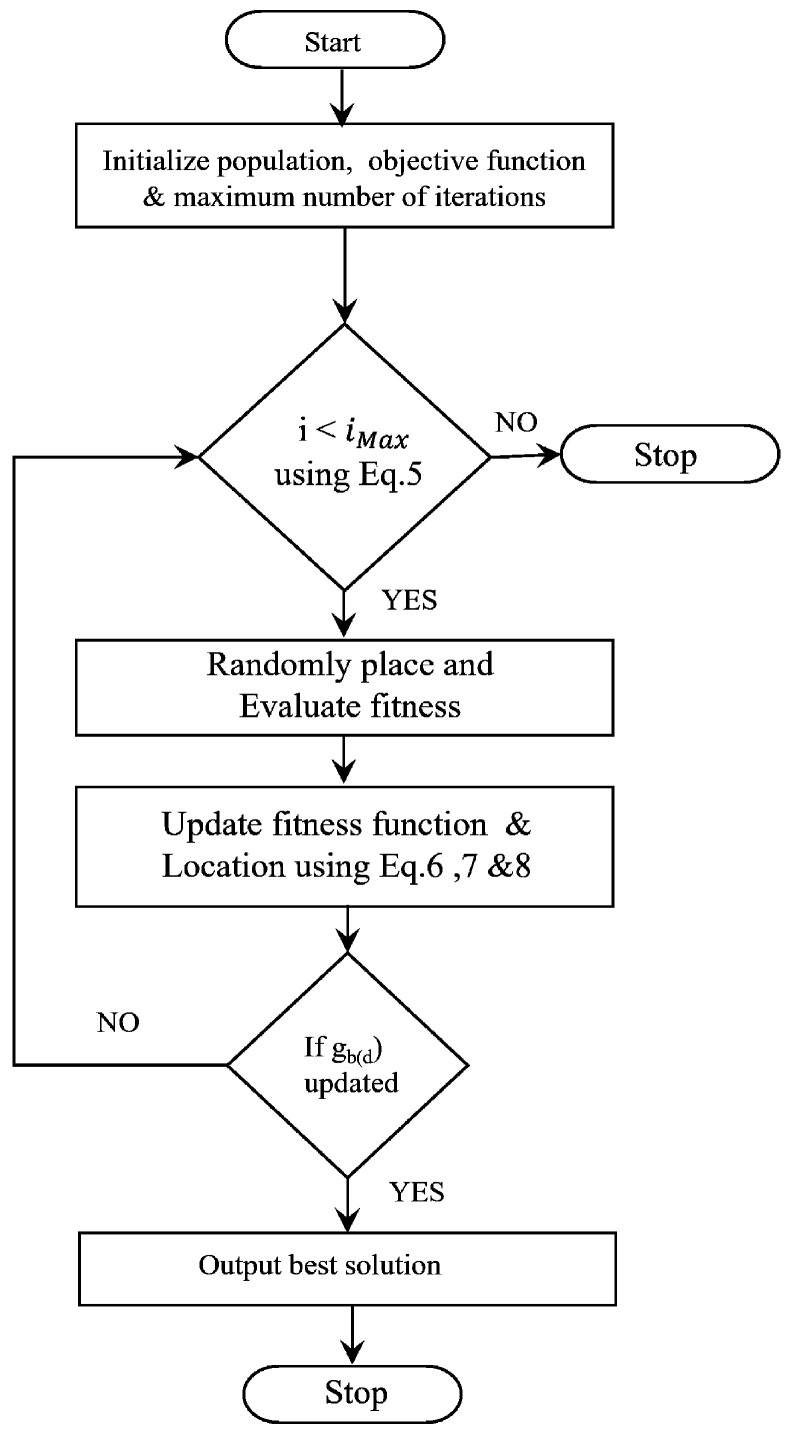
Flowchart of QKE-ABO.

ABO is an optimisation technique inspired by African buffaloes’ social and herding behaviour. The algorithm represents the three principal characteristics of buffaloes’ excellent memory, regular communication, and exceptional intelligence, which are used to ensure secure green areas around South Africa. Buffaloes can keep track of their routes through their extensive memory capacity. Finally, to denote good or bad, the second characteristic of buffaloes is their ability to communicate with one another intelligently by utilising the sounds “maaa” and “waaa”, respectively. Buffaloes rely on the parliamentary characteristics of the herd. Thus, the searching process of buffaloes considering these three features is a set of strategies organised mathematically in the proposed framework for selecting the optimal parameters. However, the min–max normalisation used for the exploitation of buffaloes slows down the convergence process [58]. Hence, in our proposed work, the quadratic kernel function was used in the exploitation phase of the buffaloes. QKE-ABO is an attempt to develop a user-friendly, robust, efficient, effective, simple-to-implement algorithm that demonstrates exceptional capacity in selecting the optimal parameters. The flowchart used to implement it is given Figure 5.

The steps of the Algorithm 1 are as follows:

Step 1: First, the population of buffaloes (i.e., the parameters that need to be optimised) is randomly initialised in the n-dimensional search space, and each individual’s fitness is evaluated and updated. Here, the fitness is evaluated based on the minimum value of parameters such as R_HOLD_TIME, RREQ jitter, NET_TRANSVERSAL_TIME, and RREP_ACK_TIMEOUT with their values initialised as in [59], and the fitness function can be expressed as in Equation (5).

(5) $$Fn\left\{pm_{i=1,2,3,4}\right\}=\sum_{i=1}^{4}\min\left(pm_{i}\right),\;\min\left(pm_{i}\right)=\left\{\hbar_{\min}<pm_{i}<\hbar_{\max}\right\}$$

where the fitness function is denoted as Fn(•), the number of parameters, such as RREQ jitter, R_HOLD_TIME, NET_TRANSVERSAL_TIME, and RREP_ACK_TIMEOUT, is illustrated by $pm_{i}$, and the minimum and maximum ranges of a corresponding parameter are expressed as $\left(\hbar_{\min},\hbar_{\max}\right)$.

Step 2: By employing African buffaloes’ two basic sounds (maaa and waaa), the exploitation and exploration of each individual are determined in the period of migration movement. The buffaloes were forced to stay in the current location by utilising the “maaa” calls. It is safe to exploit and has adequate pasture. As the current location where they are present is unsafe and lacks adequate grazing fields, the buffaloes were warned to explore the other grazing areas by utilising the “waaa” calls. Hence, the two sounds are modelled as

(6) $$z_{m}\left(d+1\right)=\phi \left(x,z_{m}\left(d\right)\right)+y_{i1}\left(g_{b}\left(d\right)-e_{m}\left(d\right)\right)+y_{i2}\left(p_{b}\left(d\right)-e_{m}\left(d\right)\right)$$

(7) $$\phi \left(z_{m}\left(d\right)\right)={\left(x^{T}z_{m}\left(d\right)\right)}^{2}$$

where $Z_{m}$ and $e_{m}$ represent the migration moves of each individual during the exploitation and exploration of the search space, the learning parameters $y_{i1}$ and $y_{i2}$ are between 0 and 1, the global best solution is specified as $g_{b}$, *d* is the current iteration, the quadratic kernel function is denoted as ϕ(•), the number of kernels is *x*, and the individual’s best solution is $p_{b}$.

Step 3: In comparison to its current position, the previously visited areas were tracked by the African buffaloes along with the solution acquired from all to alert about the most-effective position. Informed decisions were made by this search for solutions.

Step 4: The buffalo’s position is updated as

(8) $$e_{m}\left(d+1\right)=\frac{e_{m}\left(d\right)+z_{m}\left(d+1\right)}{\gamma }$$

where γ takes a value at random between (0,1), the shift towards the current position is denoted as $e_{m}\left(d+1\right)$, and $e_{m}\left(d\right)$ is the current exploration value of the buffalo. Then, the fitness is evaluated, and the best solutions are saved. Finally, the algorithm checks for the improvement of the best buffalo. After updating, the stopping criteria are evaluated. The algorithm continues until the best fitness meets the stopping criteria. Algorithm 1 shows the pseudo-code of the QKE-ABO algorithm.

Based on the adjustments made to the buffalo’s mobility during the iterations, the last optimum result is obtained. In this way, the QKE-ABO algorithm is utilised to select the optimal LOADng parameters that maximise the PDR with the minimum routing delay. Then, with the optimal parameters, efficient routing is performed using the qAB_LOADng protocol.

The vital elements for route discovery and route maintenance are as follows:

- RREQ: This has the destination’s address as the source node (SN) and is forwarded to the destination.
- RREP: This is responsible for sending a reply to the RREQ after the destination node (DN) receives the RREQ from the SN.
- RREP ACK: To confirm the reception of the RREP message, this packet is used by the LOADng router.
- RERR: The route error message is used to alert about the failure in forwarding the data.

For the data transmission, a smart grid communication network was utilised. From that network structure, multiple paths are discovered by the qAB_LOADng protocol. With the assistance of the above-mentioned control messages, all the possible solutions are discovered, and the source nodes send the data packets to the destination node via these routing paths. The discovered multiple routing paths are mathematically expressed as

(9) $$\psi_{i}=\left\{\psi_{1},\psi_{2},\psi_{3},\ldots \ldots ,\psi_{n}\right\}$$

where the routing paths are defined as $\psi_{i}$ and the nth routing path is specified as $\psi_{n}$. As for the SN to the DN, the routing paths should be consistent, highly energy-efficient, and the shortest to obtain the minimal transmission delay and energy consumption. Therefore, choosing the appropriate routing path is an essential aspect to be covered in a smart grid communication network. Therefore, the optimal routing path was discovered by using the scrambled-mutation-based Mexican axolotls optimisation (SMAx) algorithm.
**Algorithm 1** Pseudo-code of the QKE-ABO algorithm.Input: parameters of LOADng protocolOutput: optimised parameters**Begin****Initialise** population, objective function, maximum number of iterations $i_{\max}$,**While** $\left(i\leq i_{\max}\right)$       **Randomly** place buffaloes        **Evaluate** fitness of buffaloes        **Update** fitness of buffaloes:$$z_{m}\left(d+1\right)=\varphi \left(x,z_{m}\left(d\right)\right)+y_{i1}\left(g_{b}\left(d\right)-e_{m}\left(d\right)\right)+y_{i2}\left(p_{b}\left(d\right)-e_{m}\left(d\right)\right)$$
$$\varphi \left(z_{m}\left(d\right)\right)={\left(x^{T}z_{m}\left(d\right)\right)}^{2}$$       **Update** the location of buffaloes:$$e_{m}\left(d+1\right)=\frac{e_{m}\left(d\right)+z_{m}\left(d+1\right)}{\gamma }$$       **Evaluate** fitness       **If** $(g_{b}\left(d\right)$ = Updating )              **Validate** the termination criteria       **Else**              **Repeat** the updating process       **End if**              **Return** optimal parameters**End while****End**

### 3.4. Optimal Path Selection

The MAO algorithm is a meta-heuristic algorithm that has the capability of mimicking the birth, reproduction, and tissue regeneration of axolotls, as well as the way they live in the aquatic environment. The axolotl population is divided into males and females. Here, the axolotls are considered as multiple paths $\psi_{i}$ created by the protocol. The axolotl’s capacity to alter the colour of its body parts to camouflage itself and avoid predators is considered by this algorithm. In conventional MAO, the update process could easily fall into the optimum local value. In the update step, the scrambled mutation function was applied in the proposed work. The proposed SMAx algorithm’s mathematical model is derived as follows: By assigning each individual as male or female, two sub-populations are acquired from the initial population of axolotls (which is initialised randomly). This is owed to the axolotls’ development regarding their sex. They adjust the colour of their body parts toward the best-adapted individuals, which occurs after the initial transition from larvae to adult is complete.

The steps involved in the optimal path selection from the QKE-ABO is given as follows.

**Figure 6:**
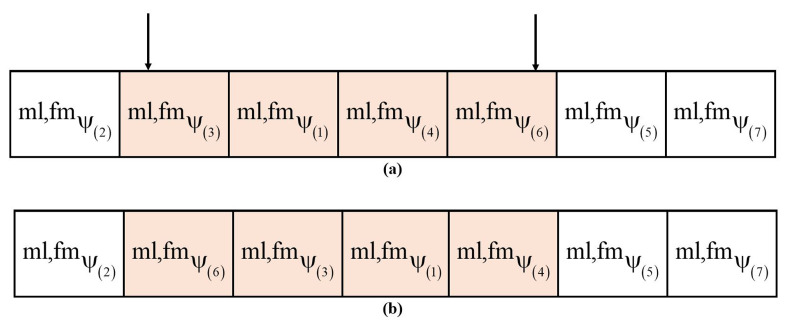
(**a**) Population positions before scrambled mutation; (**b**) Population positions after scrambled mutation.

Let ${}^{ml}\psi_{\left(bst\right)}$ and ${}^{fm}\psi_{\left(bst\right)}$ be the best-adapted male and female individuals and τ be the transition parameter for changing the colour of the body parts of the male and female axolotls. Then, the scrambled mutation is applied to the male and female populations, which is shown in Figure 6. For example, in Figure 6a, two positions are selected randomly between Positions 1 and 6, then scrambled mutation is performed between them. As is shown in Figure 6b, Position 5 is scrambled to Position 2.

A subset of individuals is chosen from the population in scrambled mutation, and their values are scrambled or shuffled. From one generation of a population to the next, genetic diversity is maintained and aids in exploitation, as well as it speeds upthe convergence. During the scrambled mutation stage, the best male and female axolotls are selected based on the objective function. Then, the remaining individuals are selected based on the inverse probability of the transition of male axolotls and female axolotls given in Equations (11) and (12). The fitness is evaluated based on the minimum distance between the source and destination nodes, as given in Equation (10).

(10) $$fit\left({}^{ml}\psi_{\left(q\right)},{}^{fm}\psi_{\left(q\right)}\right)=\left\{\tau_{\min}|\tau =\psi_{i(src)}-\psi_{i(dtn)}\right\}$$

(11) $$\zeta_{{ml}_{\psi_{\left(q\right)}}}=\frac{fit\left({}^{ml}\psi_{\left(q\right)}\right)}{\sum fit\left({}^{ml}\psi_{\left(q\right)}\right)}$$

(12) $$\zeta_{{fm}_{\psi_{\left(q\right)}}}=\frac{fit\left({}^{fm}\psi_{\left(q\right)}\right)}{\sum fit\left({}^{fm}\psi_{\left(q\right)}\right)}$$

where ζ denotes the inverse probability of male ${}^{ml}\psi_{\left(q\right)}$ and female ${}^{fm}\psi_{\left(q\right)}$ axolotls being computed using the optimisation value $fit\left({}^{ml}\psi_{\left(q\right)},{}^{fm}\psi_{\left(q\right)}\right)$ and $\tau_{\min}$ is the minimum distance between the source $\psi_{i(src)}$ and destination locations $\psi_{i(dtn)}$ of the corresponding path. Those individuals change the colour of their bodies using the random parameter as $v_{q}\in \left(0,1\right)$.

(13) $$\begin{array}{cc} \; & {}^{ml}\psi_{\left(p,q\right)}=q_{\min}+\left(q_{\max}-q_{\min}\right)*v_{q} \end{array}$$

(14) $$\begin{array}{cc} \; & {}^{fm}\psi_{\left(p,q\right)}=q_{\min}+\left(q_{\max}-q_{\min}\right)*v_{q} \end{array}$$

where the minimum and maximum values of the qth body parts are denoted as $q_{\max}$, $q_{\min}$. Hence, the best values of the males and females are updated. Injuries and accidents occur while axolotls move in the water. This may result in losing some body parts. By utilising the regeneration probability $\zeta_{\left(p.q\right)}^{\prime }$, it replaces its body parts when the loss probability is satisfied as   

(15) $$\zeta_{\left(p.q\right)}^{\prime }=q_{\min}+\left(q_{\max}-q_{\max}\right)*v_{q}$$

**Figure 7:**
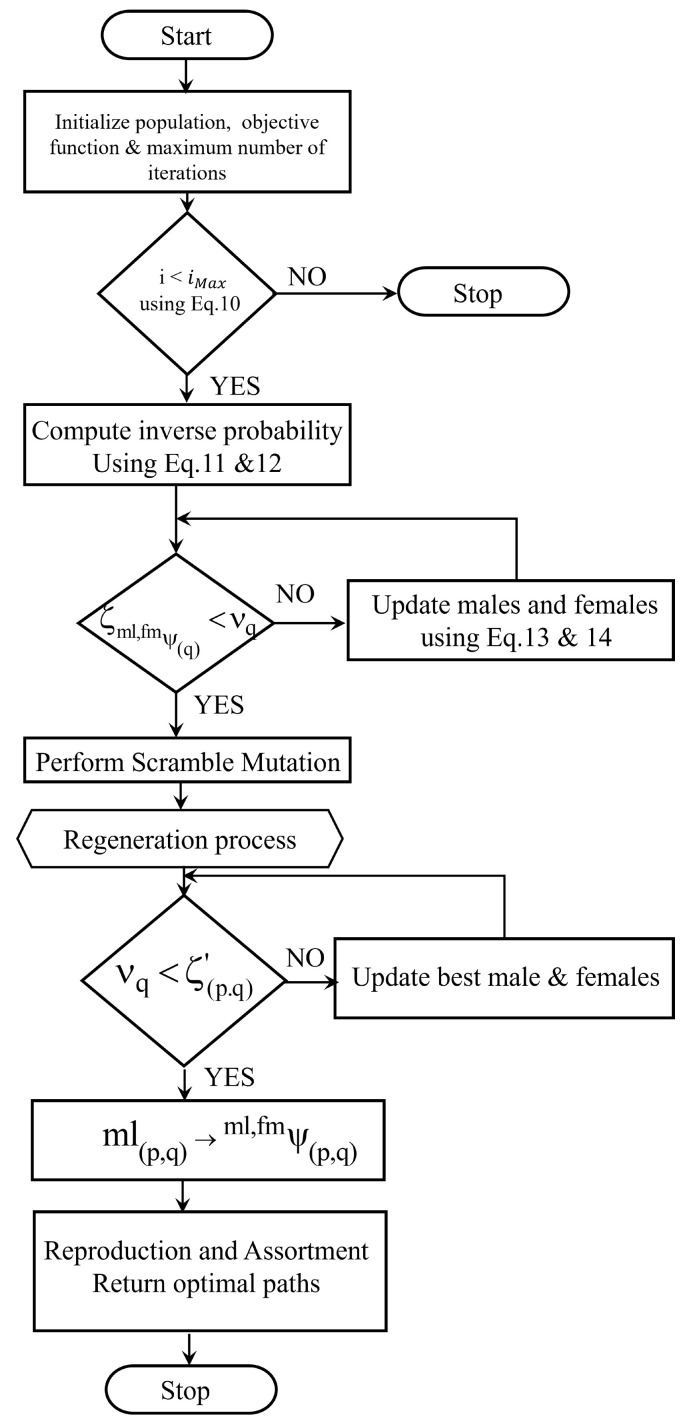
Flowchart: SMAX.

By choosing a male axolotl for each female axolotl in the population, the offspring were obtained in the reproduction phase. To start the assortment process, the male lays sperm and the female places glucose on the sperm, on which it lays its eggs, and the hatching process occurs in this phase. The young larvae are transplanted to a better condition if they are better fit. Algorithm 2 shows the pseudo-code of the SMAx algorithm. The flowchart used to implement it is given in Figure 7.

In this way, the optimal path was selected between the source and destination by applying the SMAx algorithm. The optimal path refers to the path that has the maximum energy level with the minimum distance. Over this optimal routing path, the data packets were forwarded from the source node to the destination node.
**Algorithm 2** Pseudo-code of SMax algorithm.Input: multiple created pathsOutput: optimal paths**Begin**       **Initialise** population, objective function, maximum number of iterations $i_{\max}$,        **While**$\left(i\leq i_{\max}\right)$                      transition from larvae to adult       **Select** the best male and female axolotls       **Compute** inverse probability $\zeta_{{}^{ml,fm}\psi_{\left(q\right)}}$       **If** $\left(\zeta_{{}^{ml,fm}\psi_{\left(q\right)}}<\nu_{q}\right)$               **Perform** scrambled mutation       **Else**              **Update** ${}^{ml,fm}\psi_{\left(p,q\right)}=q_{\min}+\left(q_{\max}-q_{\min}\right)*\nu_{q}$       **End if**       //regeneration process       **For each** (${}^{ml,fm}\psi_{\left(p,q\right)}$)       **If** $\left(\nu_{q}<\zeta_{\left(p.q\right)}^{{}^{\prime }}\right)$       $ml_{\left(p,q\right)}\leftarrow {}^{ml,fm}\psi_{\left(p,q\right)}=q_{\min}+\left(q_{\max}-q_{\min}\right)*\nu_{q}$       **Else**              **Update** ${}^{ml,fm}\psi_{best,q}$       **End if**       //reproduction and assortment       **Select** suitable male       **Obtain** two eggs       **Sort** ${}^{ml,fm}\psi_{p,q}$ and two eggs**End while****Return** optimal paths**End**

### 3.5. LoRa Gateway

A gateway with a LoRa connection may aid in end node communication. The data are stored and maintainedby the users in the gateway, which serves as a bridge between the end nodes and the network servers. There was a concentrator board that can receive and forward the LoRa packets to the server in the LoRa gateway. For control purposes, reverse communication from the server to the nodes was allowed by it. It could gather all the data received from the nodes even though the gateway had no data processing capabilities. Then, they were relayed to the server for processing.

## 4. Results and Discussion

To satisfy the stringent QoS requirement of AMI applications, the proposed model’s performance is analysed in this section. Using the Cum_LEACH and QKE-ABO path selection techniques, the proposed SG communication mode was simulated in the MATLAB platform. Table 3 shows the simulation parameters associated with the proposed model.

### Performance Analysis

Performance matrices such as the energy value, energy consumption, number of operating nodes, number of dead nodes, and the node lifetime were calculated for the proposed algorithm and tested with the existing LEACH, DEEC, and SEP protocols. The energy value gives the information about the remaining energy in the node after transmitting the data for specific runs.

**Figure 8:**
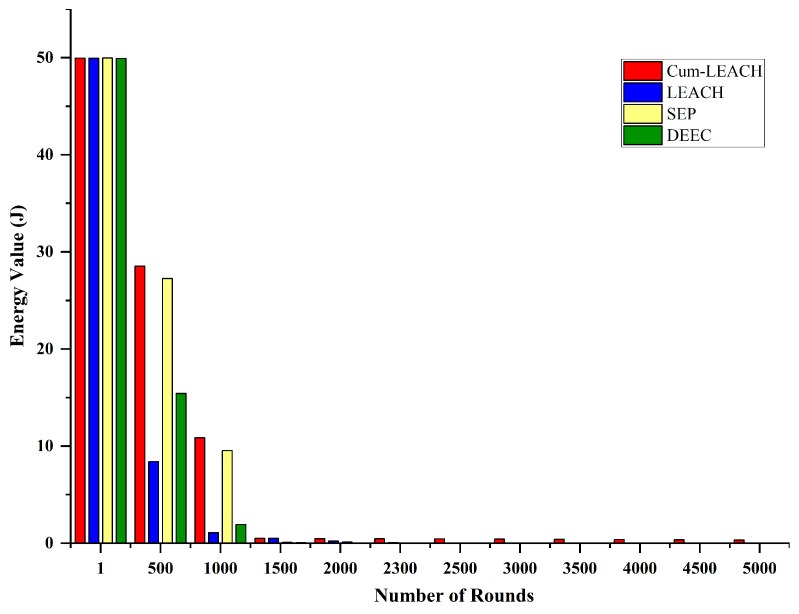
Performance analysis of the proposed and existing protocols based on the energy value.

From Table 4, it can be noted that, as the number of runs increased, the energy value of the proposed method decreased. This indicates an increase in dead nodes and a decrease in operating nodes. Compared to the existing methods, the energy value retained by the proposed method was higher than the existing methods for the same number of runs, as shown in Figure 8. For instance, the protocol was implemented for 500 runs. The proposed Cum_LEACH method had an energy value of 28.25 J, whereas the energy value of LEACH was 8.39 J; the energy value of DEEC was 27.24 J; the energy value of the SEP was 15.43 J. This indicates that the proposed Cum_LEACH retained a higher energy value compared to the existing methods.

The energy consumption analysis for the proposed and existing methods is presented in Table 5. The energy consumed by the proposed Cum_LEACH method was the least at 500 runs. Even after 5000 runs, the total energy of the nodes was not totally consumed, whereas for the other methods, the energy was fully consumed. Thus, from the analysis, it can be inferred that when the Cum_LEACH protocol was used in conjunction with LoRa nodes, the energy consumption was less when compared to other protocols.

**Figure 9:**
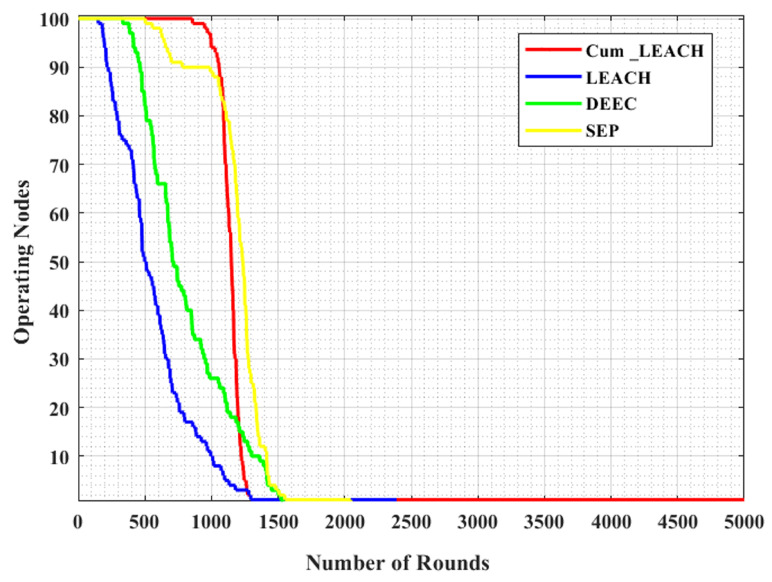
Number of operating nodes vs. number of rounds for various CH selections.

The number of operating nodes for the different number of runs is shown in Figure 9. The time until the first sensor’s energy runs out is known as the network lifetime. The results showed that the number of active nodes after 500 runs was 100 with the proposed Cum_LEACH protocol, but in the cases of LEACH, DEEC, and the SEP, the number of active nodes was 50, 82, and 99, respectively. After 1000 runs, the number of active nodes was 99, 10, 26, and 89 for the Cum_LEACH, LEACH, DEEC, and SEP protocols, respectively. Similarly, after 2500 runs, the proposed system had some active nodes, but the other systems had no active nodes, as shown in Table 6. It was also observed that the lifetime of the proposed system was extended up to 5000 runs, whereas the existing protocols’ network lifetime was 2390, 1536, and 2046 runs (for LEACH, DEEC, and the SEP, respectively). Compared with the existing models, the proposed method Cum_LEACH had a higher number of operating nodes.

**Figure 10:**
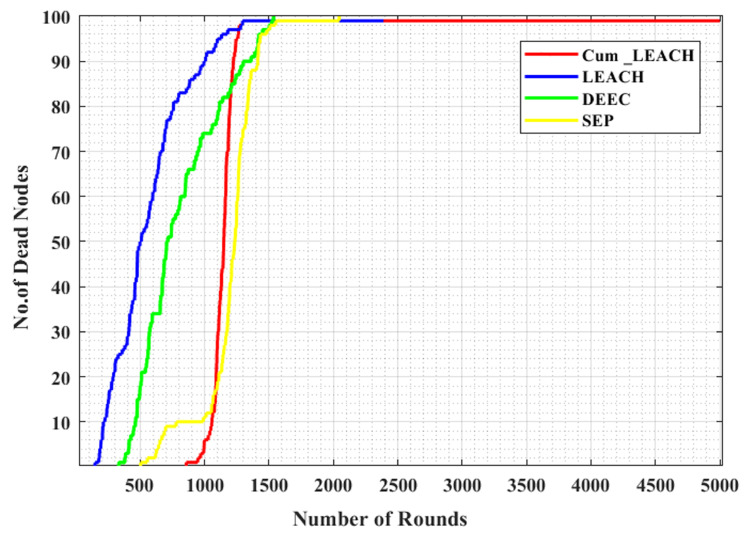
Number of dead nodes vs. number of rounds for various CH selections.

**Figure 11:**
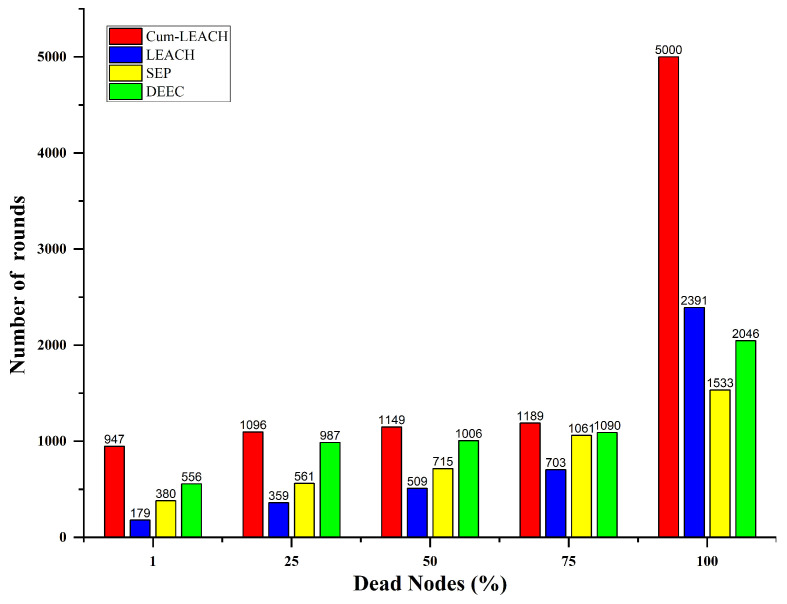
Number of rounds vs. percentage of dead nodes for various CH selections.

From Figure 10, it is observed that the number of dead nodes identified when the Cum_LEACH protocol was used for CH selection was much less when compared to the other standard protocols. With the Cum_LEACH protocol, the first node died after 850 runs, whereas for LEACH, DEEC, and the SEP, the first node died within 150, 335, and 504 runs, respectively. Similarly, after 2500 runs, for the existing protocols, all the nodes were dead, but the proposed system still had some active nodes. Therefore, this shows that, for each run, the number of dead nodes in the SG when tested with the proposed protocol was much less compared to the number of dead nodes using the other protocols. For the proposed protocol Cum_LEACH implemented for 5000 runs, the runs at which the first and last node died are given in Table 6. The percentage of dead nodes vs. the total number of runs is given in Figure 11. From this figure, it can be noted that 50% of the nodes were dead at 1149 rounds, whereas for the LEACH, SEP, and DEEC protocols, the number of runs was 509, 715, and 1006, respectively. This clearly helped in the extension of the network lifetime and, hence, helped maintain the network for a longer duration.

**Figure 12:**
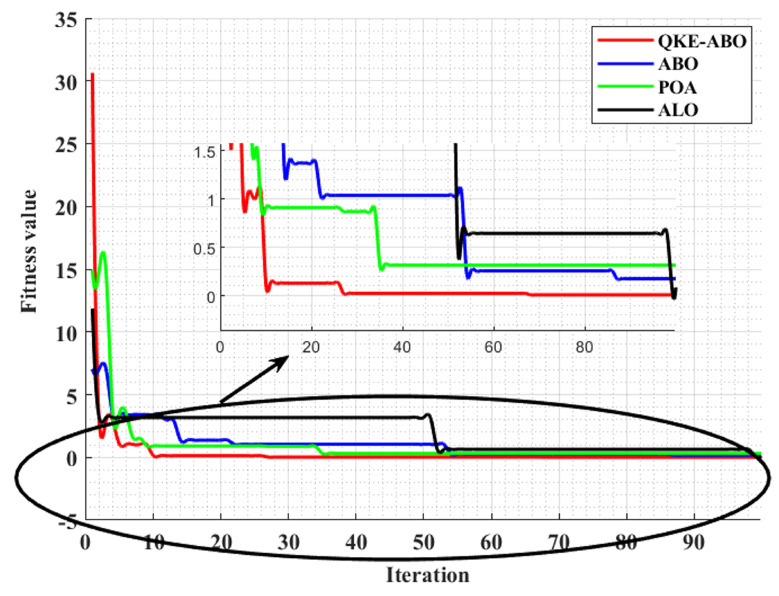
Comparative analysis of the QKE-ABO algorithm with existing algorithms.

The effectiveness of QK_ABO in generating multiple optimal paths once the CHs were selected is presented using the fitness value plot in Figure 12. A comparative analysis of the convergence of the proposed QKE-ABO and African buffalo optimisation (ABO), pelican optimisation algorithm (POA), and ant lion optimisation (ALO) algorithms is presented with the fitness value as a function of the optimisation iterations.

**Figure 13:**
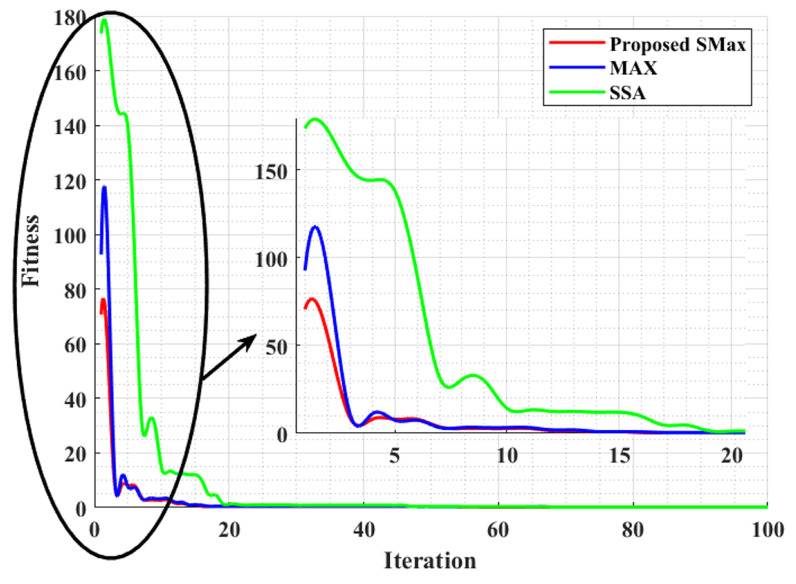
Comparative analysis of the proposed SMAx algorithm with existing methods.

The optimisation outcomes obtained regarding the objective function’s number of evaluations are exhibited in Figure 10. The convergence to the optimal solution is exhibited by the net minimal value. In comparison to the existing models, the proposed QKE-ABO algorithm converged rapidly toward the minimal value. Hence, the proposed QKE-ABO method is more efficient. The full convergence curves of the proposed SMAx and the existing Mexican Axolotls Optimisation (MAX) and Sparrow Search Algorithm (SSA) are exhibited in Figure 13. The iterative process’s convergence was ensured by the parameter ranges. When compared to the existing models’ search process, the proposed SMAx algorithm converged to an approximate result after fewer iterations, exhibiting the model’s better performance.

## 5. Conclusions

In this paper, a novel cluster head selection protocol called Cum_LEACH was proposed. A novel QKE-ABO optimisation process was proposed to generate multiple optimal paths between sender and receiver nodes when they are placed far apart. Next, a modified MAX algorithm called the SMAx algorithm was proposed to select the best path among the paths specified by the QKE-ABO algorithm. The performance of the Cum_LEACH algorithm was assessed using parameters such as the energy value, dead nodes, and operating nodes as a function of the number of times the data were transmitted by the network, called runs. The performance of the proposed Cum_LAECH was compared with the standard LEACH, DEEC, and SEP algorithms. It was observed that the energy value of the nodes was nonzero in the case of Cum_LEACH, whereas the energy value was zero in the case of the LEACH, DEEC, and SEP algorithms when the number of runswas 5000. At 1000 runs, the energy values were 10.86 J, 1.08 J, 9.53 J, and 1.92 J for the Cum_LEACH, LEACH, DEEC, and SEP algorithms, respectively. The number of dead nodes is another parameter of interest that gives us information about the longevity of a network. It was also shown that the number of dead nodes was very low in the case of the Cum_LEACH protocol compared to the other standard protocols when the number of runs was 1000. At 5000 runs, all the nodes were dead in the case of the Cum_LEACH protocol, whereas in the case where the LEACH, DEEC, and SEP protocols were used, all the nodes were dead at 2390, 2046, and 1536 runs. In the case that the number of active nodes was considered as a performance parameter, Cum_LEACH performed better compared to the other standard protocols. Once the CH selection was performed, for the transmission process, multiple paths were created using the proposed QKE-ABO algorithm, whose performance was compared with the other optimisation algorithms using the fitness value parameter. The fitness value converged after 28 iterations in the case of the QKE-ABO algorithm, whereas the value converged after 35, 53, and 53 iterations for the POA, ALO, and ABO algorithms, respectively. Once the paths were created, to select the best path, the SMAx algorithm was proposed. Its performance was assessed using the fitness value and was compared with the MAX and SSA algorithms. For the SMAx algorithm, the number of iterations taken for the fitness value to converge was 15, whereas it was 18 in the case of MAX and 19 in the case of the SSA algorithm. There was no greatly significant improvement by the proposed SMAX algorithm when compared to the MAX and SSA algorithms. The computational complexity and speed of convergence involved in the proposed algorithms in comparison to the existing algorithms should be taken up as future work, as well as the feature extraction of the AMI sensor nodes using advanced neural networks to detect the potential for intrusion into the AMI, which is predicted over a novel neural network classifier, and for improved performance, along with the development of lightweight security schemes to ensure the integrity and confidentiality of the metring data in SGs.

## Figures and Tables

**Table 1:** Comparison of energy-efficient WSN routing techniques used for smart grid applications.

Reference	Protocol	Description	Limitations
[29]	Improved-adaptive-ranking-based energy-efficient opportunistic routing protocol (I-AREOR)	An energy-balanced clustering protocol to apply the green IoT to smart cities	Limited to the energy efficiency problem in WSNs; does not consider factors such as the latency, throughput, and reliability of data transmission
[30]	Path operator calculus centrality (SPOCC)	Analysis of the E.C. in SG WSNs utilising an SPOCC-centred HSA-PSO algorithm	Limited to SG outdoor transmission; security enhancement was identified as an impending factor, but not addressed in this paper
[31]	Fuzzy multi-criteria clustering and bio-inspired energy-efficient routing (FMCB-ER)	Hierarchical WSN protocol with FMCB-ER	As the complex algorithm, EPO, for route optimisation from the CHs to the sink nodes requires a large amount of computational power, it is impossible on low-power sensors
[32]	Ticket-based routing (TBR)	Ticket-based QoS routing optimisation utilising G.A. for WSN applications in SGs	Tested on fixed sensor nodes; the genetic algorithm was applied only to the source sensor nodes
[33]	BEST-MAC	Enhanced BEST-MAC protocol for WSN utilising optimum CH selection	It is not capable of handling adaptive data rates; it considers limited parameters to improve the lifetime of sensor networks
[34]	Adaptive quorum-centred AQ-MAC protocol	A quorum-centred energy-efficient MAC for WSNs	Suitable only for data collection system; limited in scalability due to the low duty cycle
[35]	Quorum-centred MAC (QMAC) protocol	A quorum-centred energy-saving MAC protocol for WSNs	Limited to a many-to-one communication model; it relies on synchronisation among sensors; any discrepancies affect its performance

**Table 2:** Notations and definitions.

Symbol	Definition	Symbol	Definition
*ℵ*	Set of sensor nodes	${ℵ}_{i}$	ith sensor node
*ℓ*	Random number	$\Omega_{cdf}$	CDF
α	Probability	$r_{cu}$	Current round is modelled
λ(i)	Threshold	${\;}^{g}{ℵ}_{i}$	Nodes do not contribute as CHs
${ℵ}_{CH(i)}$	Number of CHs selected	Fn(•)	Fitness function
$\left(\hbar_{\min},\hbar_{\max}\right)$	Minimum and maximum range	$y_{i1}$, $y_{i2}$	Learning parameters
$z_{m}$ and $e_{m}$	Exploration and exploitation	$g_{b}$	Global best solution
*d*	The current iteration	ϕ(•)	Quadratic kernel function
$p_{b}$	Individual’s best solution	$e_{m(d)}$	Current exploration values
$e_{m(d+1)}$	Shifted current position	$\psi_{i}$	Routing paths
$p_{b}$	Individual’s best solution	$e_{m(d)}$	Current exploration values
γ	Random number (between 0 and 1)	${}^{ml}\psi_{\left(bst\right)}$, ${}^{fm}\psi_{\left(bst\right)}$	Best-adapted male and female
τ	The transition parameter	ζ	Inverse probability
$\zeta_{\left(p.q\right)}^{\prime }$	Regeneration probability	$v_{q}\in \left(0,1\right)$	Random parameter (between 0 and 1)

**Table 3:** Simulation parameters.

Name	Value
Target area	100 m × 100 m
Number of nodes deployed	100
Initial energy of node	0.5 J
Energy transfer for each bit	50 nJ/bit
Energy consumption of signal amplification in free space	10 pJ/bit/m${}^{2}$
Energy consumption of signal amplification in multipath	0.0013 pJ/bit/m${}^{2}$
Energy consumption of data fusion	5 nJ/bit/packet
Control packet length	200 bits
Packet length	6400 bits
Maximum number of running rounds	5000

**Table 4:** Performance analysis of the proposed and existing protocols based on the energy value (Joules).

No. of Rounds	Cum_LEACH	LEACH	SEP	DEEC
1	49.95	49.93	49.96	49.90
500	28.52	8.39	27.24	15.43
1000	10.86	1.08	9.53	1.92
1500	0.48	0.49	0.08	0.04
2000	0.46	0.21	0.10	0.00
2500	0.44	0.00	0.00	0.00
5000	0.33	0.00	0.00	0.00

**Table 5:** Performance analysis of the proposed and existing protocols based on energy consumption (Joules).

No. of Rounds	Cum_LEACH	LEACH	SEP	DEEC
1	0.04	0.07	0.02	0.08
100	4.35	11.67	2.23	5.41
500	21.74	41.86	25.14	27.36
1000	43.39	48.95	44.58	37.53
1500	49.52	49.79	48.62	39.15
2000	49.54	49.79	49.64	49.15
5000	49.67	50.00	50.00	50.00

**Table 6:** Iteration after which the first and last dead node were observed.

No. of Rounds	Cum_LEACH	LEACH	SEP	DEEC
First node dies	853	150	504	335
Last node dies	5000	2390	2046	1536

## Data Availability

Not applicable.
